# The power of corporate control in the global ownership network

**DOI:** 10.1371/journal.pone.0237862

**Published:** 2020-08-27

**Authors:** Takayuki Mizuno, Shohei Doi, Shuhei Kurizaki

**Affiliations:** 1 Information and Society Research Division, National Institute of Informatics, Tokyo, Japan; 2 School of Political Science and Economics, Waseda University, Tokyo, Japan; Unviersity of Burgundy, FRANCE

## Abstract

As passive investment through index funds and Exchange Traded Funds (ETFs) has become pervasive, the structure of corporate control in the global capital market is more complex than before. We propose a new model and calculation algorithm to measure a shareholder’s power to control corporations in the global economy. Our method takes into account how fragmented voting rights attached to dispersed ownership may be consolidated to generate corporate control. Analyzing the ownership holdings in 49 million companies worldwide by 69 million shareholders in 2016, we find that the landscape of global corporate control appears differently if we adequately evaluate indirect influence via dispersed ownership. In particular, a larger portion of corporate control appears to be concentrated in the hands of sovereign governments than has been recognized before. Yet, such governmental capacities are “hidden” if we use the conventional method. Moreover, financial institutions appear to not be as powerful as emphasized before. These results point to important financial and political risks both for scholars and policymakers.

## Introduction

Understanding the structure of corporate control in the global capital market is increasingly important for managing financial and political risks, upstream and downstream alike. To achieve their socially responsible investments, for example, investors must identify to which corporations, and their economic activities, her “responsibility” extends downstream through the ownership network. Larry Fink, the chairman and CEO of the world’s largest asset manager BlackRock, vows to divest from thermal coal producers and other entities with a high sustainability-related risk [1]. But how does he know that investments in a low-carbon company do not trickle down to a company that invests in fossil fuels? Financial and related risks also come from upstream. For example, the State Grid Corporation of China, which is ultimately owned by the Chinese government, owns 40% of the shares of the National Grid Corporation of the Philippines. Thus, Philippine senators raise concerns that the country’s “power grid is under the full control of the Chinese government and could be shut off in time of conflict” [2].

A growing literature offers models and calculation algorithms to measure the structure of corporate control in ownership networks [3, 4]. Gambarelli and Owen were pioneers in modeling indirect control of corporations in a complex ownership network [5], and Crama and Leruth proposed an algorithm to deal with cyclic shareholdings [6]. While most previous applications were limited to particular subsets of the global ownership network, Vitali et al. were the first to analyze corporate control at the global level [7].

With the rise of passive investment through index funds and Exchange Traded Funds (ETFs), however, existing models and algorithms in the literature have become less adequate for measuring corporate control. Recent studies suggest a passive investment approach facilitates the concentration of corporate control in the hands of large financial institutions as well as the decentralization of ownership in separate funds. Fichtner et al. [8] report that in 2015 the Big Three asset management firms—BlackRock, Vanguard, and State Street—combined were the largest owner in 88% of the S&P 500 companies in the United States, but their ownership stakes rarely go beyond 10%. Similarly, Bracaccio et al. [9] report increasing centralization of capital in recent years, while Aminadav and Papaioannou [10], studying corporate ownership around the world, show that more than 53% of listed firms in 2012 are widely held with no shareholder having voting rights exceeding 20%. Against the backdrop of the growing concentration of corporate control resulting from the rise of indexed and exchange traded funds, it is challenging both theoretically and empirically to identify how shareholders (and fund managers) could orchestrate their voting rights to influence corporate decision-making. In particular, the possibility of fragmented voting rights being dispersed among equity shares is less understood, especially in relation to its impact on corporate governance.

We propose a new model and a calculation algorithm to measure how a shareholder possesses the capacity to directly or indirectly impose its will on the management decision-making in the target company. Our model and algorithm explicitly take into account a recent investment practice, in which fragmented voting rights attached to dispersed ownership may be consolidated to generate corporate control. Unlike previous studies, our empirical analysis is not limited to any particular type of corporations, sector of industry, or geographical region. It encompasses individual, governmental, and corporate shareholders in any type of company in the global capital market.

The remainder of this paper proceeds as follows. In the next section, we briefly review related literature to clarify our contributions. In Section 3, we present a model that describes the types of indirect ownership that an adequate measure of corporate control must address and propose a new measure, *Network Power Index* (NPI). Section 4 describes our approximation algorithm for the NPI based on a Monte-Carlo method. We then analyze the global ownership network and corporate control therein using our model and algorithm in section 5. The final section gives conclusions with implications for future research.

## Literature review

The study on corporate ownership and control has attracted scholars from various fields ranging from corporate finance to operations research and political economy. It is beyond the scope of this study to provide a full-fledged review of this voluminous literature. We shall limit our review to the most closely related literature, with the objective of clarifying our contribution.

One of the key achievements in the literature is the development of theoretical models and computational methods to measure indirect ownership and control. A shareholder’s ownership influence extends through the network of ownership relations, and it is implausible that the influence stops at the immediate firms that the shareholder invests in. In La Porta et al.’s [11] study that identifies ultimate owners and their corporate control around the world, they take into account how corporate control is channeled through networks by sequencing a series of direct control by transitivity (which they call control chain). Brioschi et al. [12] proposed the concept of “integrated network” to measure such control influences in terms of corporate ownership (see also [13]). In Vitali et al.’s [7] large scale empirical investigation into the structure of corporate control that explicitly takes into account its network structure, they propose the concept of *network control* to measure the economic values of corporate control a shareholder can influence through a sequence of direct ownership paths. With a few exceptions [14], the literature mostly analyzes indirect ownership and corporate control.

Identifying control is challenging and Aminadav and Papaionnou [10] identify three areas of difficulty. The first difficulty relates to sample size. Because an analysis of the global ownership network is computationally demanding, previous studies have typically worked with samples that fit the purpose of their studies. Related literature in operations research and cooperative game theory tends to focus on a small sample such as the firms listed on the Seoul Stock Exchange [6] or three specific medical firms [15]. On the other hand, previous studies in corporate finance and political economy also confine their analysis to firms of specific types in order to address problems in empirical inference and computation. For example, an influential work in corporate finance by La Porta et al. focus on the firms with $500 million market capitalization [11] and a recent work in political economy analyze only firms with revenues greater than $10 million [14]. Similarly, studies at the global level also focus on specific types of corporations, where Vitali et al. [7] limit their analysis to transnational corporations and Babic et al. [14] focus on firms owned directly by state governments or state-owned entities that invest transnationally, excluding the entities solely investing domestically. Most other studies focus on specific regions or counties, ranging from East Asia [16] to Western Europe [17], Persian Gulf countries [18], emerging markets [19], Belgium [20], Britain [21, 22], and Germany [23].

The second challenge in identifying control discussed by Aminadav and Papaionnou [10] is heterogeneity in the investment environment. While the distribution of the market capitalization of the firms is widely skewed, the corporate finance literature also stresses the importance of country-specific legal institutions as important determinants of the structure of corporate control.

The third challenge is the selection of the threshold of shares used to identify corporate control. The choice of the cutoff is likely to affect the results one would obtain regarding the distribution of control, and previous studies differ in this regard. The most common threshold is a simple majority (requiring at least 50% of voting rights) [10, 14, 20–24]; other cutoffs used in the literature are 5% [25, 26], 10% [27], 20% [10, 11], and 25% [19] for various research purposes and rationale.

An alternative to adopting a simple cutoff model to identify control is a game-theoretic method, called the Shapley-Shubik power index [28], that was originally proposed to study the potential to influence a collective decision-making in a weighted voting game. The literature is split on the usefulness of the Shapley-Shubik power index in computing voting power and the structure of corporate control in the ownership network [4, 6, 21, 22], partly because it has not been widely tested in the corporate finance literature [23] and because unlike political science and cooperative game theory that often study a very well-structured decision-making body, corporate control often involves a heterogeneous structure of distribution of stakeholders [10]. In particular, scholars have raised concerns over the adequacy of the Shapley-Shubik power index and its related measures in the presence of a cyclical shareholding structure [4, 15].

One of the advantages of the Shapley-Shubik power index, however, is that it uses a relative, as opposed to absolute, number of voting rights to map equity ownership to corporate control. As we shall demonstrate in this article, the Shapley-Shubik power index allows for measure control in firms (or in weighted voting games more generally) with multiple stakeholders and/or dispersed ownership and coalitions of stakeholders [10].

Finally, our study also shares with La Porta et al. [11] and Babic et al. [14] a focus on the role played by political actors such as state governments. La Porta et al. reported two decades ago that a non-trivial portion of large firms in advanced economies are controlled by the state governments. Babic et al. claim that “state-led foreign investment is more than just a normal FDI transaction’’ and suggest that those cross-border state investments are not only intended for purely capital-gains but also for “strategically motivated, controlling investments” (p. 434). While our study also reports that some state governments around the world have substantial capability to control corporations, we remain agnostic about the motives behind investment decisions by governments.

## Model

Consider a *network* (*N*, *x*_*ij*_) consisting of a set of companies and their shareholders *N* = {1, 2, …, *n*}, where *i* ∈ *N* indexes shareholders (be it individual, corporation, or government) and *j* ∈ *N* indexes companies whose share is owned, directly or indirectly, by *i*. An *ownership linkage* *x*_*ij*_ ∈ [0, 1] is given by the percentage of the shares of company *j* owned by each investor *i*, where the vector of company *j*’s stakes is denoted by *x*_*j*_ = (*x*_1*j*_, …, *x*_*nj*_). We say *i* has *ownership* in company *j* if *i* owns non-zero equity stakes of *j*, or if *x*_*ij*_ > 0.

A shareholder denoted by *k* ∈ *N* is a third-party to the *ij* relationship. Suppose that *i* has no direct ownership of *j* (i.e., *x*_*ij*_ = 0), but *k* has ownership in *j* and *i* has ownership in *k* (i.e., *x*_*ik*_ > 0, *x*_*kj*_ > 0). Then, by transitivity, *i* indirectly owns *j* through *k*’s ownership in *j* (i.e., ${\bar{x}}_{ij}>0$). In this case, *i* is *j*’s *ultimate owner*.

The size of equity ownership does not immediately translate into the power of managerial control. We derive a *control linkage* *y*_*ij*_ ∈ {0, 1}, from *x*_*ij*_ and company *j*’s *quota* (i.e., the minimum number of votes required to make a decision) denoted by *q*_*j*_ ∈ (0, 1] such that *y*_*ij*_ = 1 if *x*_*ij*_ > *q*_*j*_; and *y*_*ij*_ = 0 otherwise.

**Fig 1:**
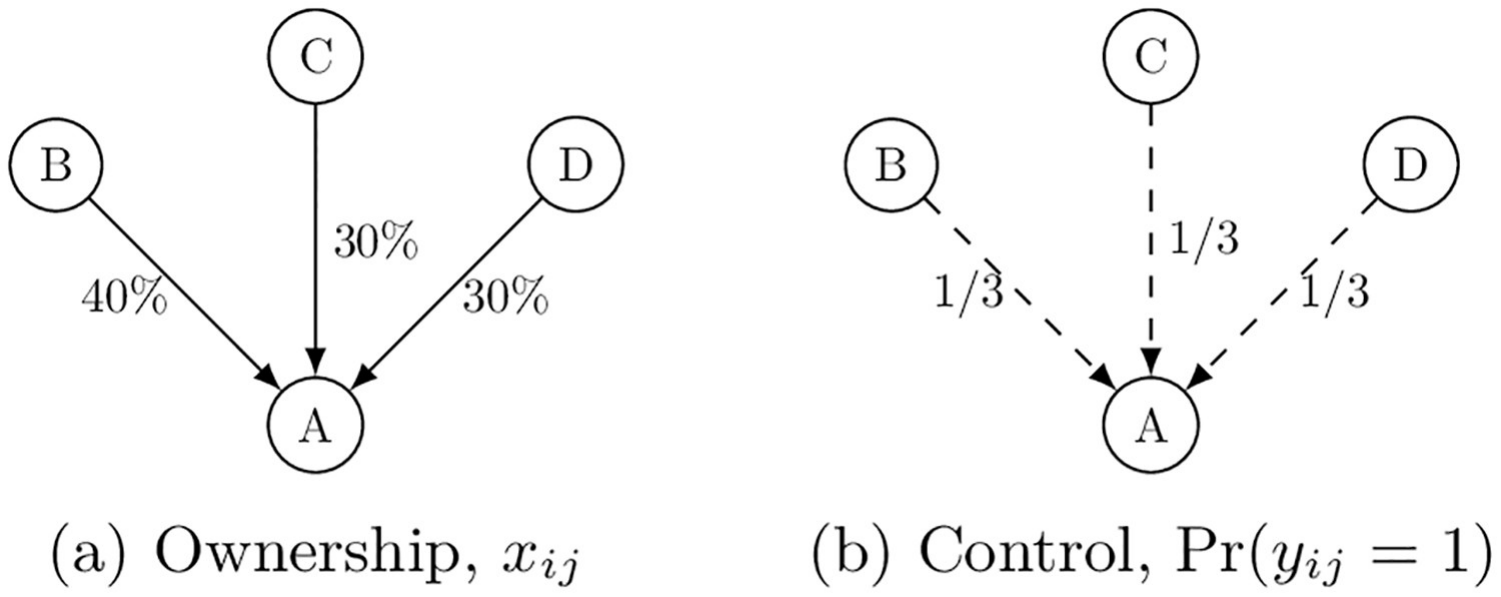
Direct ownership & control.

In the stylized example of direct ownership depicted in Panel (a) of Fig 1, three shareholders, *B*, *C*, and *D* respectively own 40%, 30% and 30% shares of company *A*. Assuming simple majority rule, *q*_*A*_ = 1/2, for *A*’s decision-making, none of the shareholders alone seizes full control of *A*, or *y*_*iA*_ ≠ 1 since *x*_*iA*_ < *q*_*A*_ for *i* ∈ {*B*, *C*, *D*}. Yet, because each of these shareholders may consolidate (proxy) voting rights to form a winning coalition, we follow Shapley and Shubik [28] to assume that they may randomly form such a coalition out of a permutation of possible combinations. This gives each of these shareholders an equal probability to prevail in *A*’s decision-making, so that for *i* ∈ {*B*, *C*, *D*} we have Pr(*y*_*iA*_ = 1) = 1/3 (Panel (b) of Fig 1).

**Fig 2:**
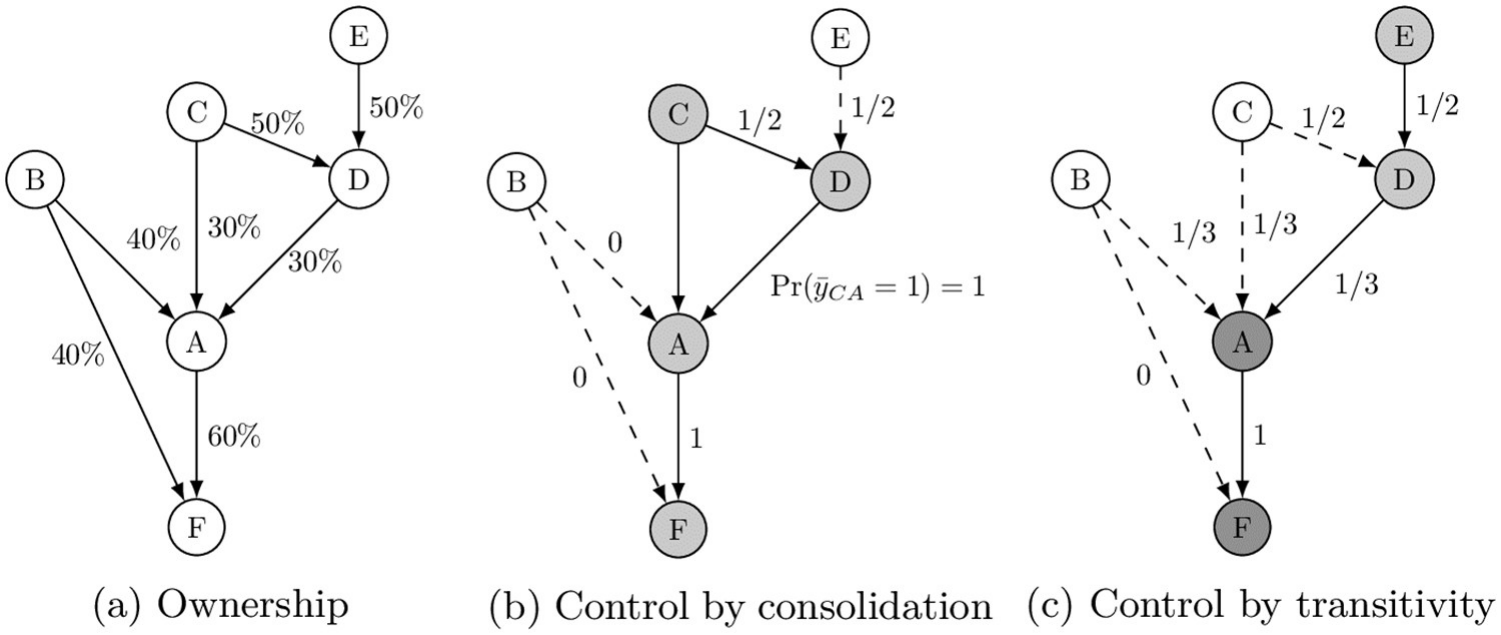
Indirect ownership & control.

Now consider an extended network of ownership that adds *E* and *F* (Panel (a) Fig 2), where investors *C* and *E* each have a 50% share of company *D*’s equity, and *A* and *B* respectively own 60% and 40% of *F*’s equity stakes. Since both *C* and *E* fall short of dominating *D*’s decision-making (e.g., *x*_*CD*_ < *q*_*D*_), each can seize control of *D*’s decision making with Pr(*y*_*iD*_ = 1) = 1/2 for *i* ∈ {*C*, *E*}.

If *C* happens to prevail in *D*, as shown in Panel (b) of Fig 2 it would indirectly control *D*’s 30% share of *A*’s equity on top of its direct ownership of the 30% share. Consolidating its direct and indirect ownership, *C*’s combined share of *A* is given by ${\bar{x}}_{CA}=x_{CA}+x_{DA}=60\%$, achieving a majority ${\bar{x}}_{CA}>q_{A}$. Consequently, *C* obtains the power to fully control *A*’s decision-making, or ${\bar{y}}_{CA}=1$, despite the fact that *B* has more shares of *A*’s equity stakes than *C* does. Furthermore, since *A* owns 60% of *F*’s shares, *C* also obtains the power to indirectly control *F* by transitivity, through its control of *D* and *A*.

If *E* prevails in *D*, as shown in Panel (c) of Fig 2, it cannot control *A* with certainty because ${\bar{x}}_{EA}<q_{A}$. Instead, *E* can only control *A* with probability 1/3, and when it does, *E* would also be able to exert its control to *F* by transitivity since *A* holds the majority shares of *F*. That is, *E* obtains the full control of *F* indirectly with probability 1/2 × 1/3 = 1/6 as part of what La Porta et al. call a control chain [11]. However, if *B* or *C* happens to prevail in *A*, precluding *E*’s control of *A*, then *E* cannot extend its influence to *F*.

### Measuring the power of corporate control

The conventional way to identify corporate control a shareholder *i* may obtain through its direct and indirect paths of ownership is to integrate the sequence of its direct controls from *i* to *k* (i.e., *y*_*ik*_ = 1) and *k* to *j* (i.e., *y*_*kj*_ = 1). This approach amounts to what we call the indirect control by transitivity in Panel (c) of Fig 2, but falls short of identifying indirect control by consolidation as in Panel (b). Take the concept of *network control* by Vitali et al. [7] as an example. In our example of indirect ownership in Fig 2, *network control* for *C* is 0, since *C* does not have full control over any other entity in this network. This would underestimate the influence exercised by large institutional investors with dispersed and fragmented ownership.

Another, more intuitive measure for corporate influence is what we call the *Network Shareholding Ratio* (NSR), which is given by the product of the shares of direct ownership, *x*_*ik*_ ⋅ *x*_*kj*_. This is similar to the weighted measure known as “integrated ownership” proposed by Brioschi et al. [12] that uses direct investment to construct indirect ownership links. In our example, *C*’s NSR in *F* is given by *x*_*CD*_ ⋅ *x*_*DA*_ ⋅ *x*_*AF*_+ *x*_*CA*_ ⋅ *x*_*AF*_ = 0.5 × 0.3 × 0.6 + 0.3 × 0.6 = 0.09 + 0.18 = 0.27. The NSR is useful for characterizing shareholders’ liquid ownership without control, where their ability to influence networked capital markets can be felt through their divestment depresses the share price rather than through direct engagements and publicity campaigns.

We introduce a new measure, the *Network Power Index* (NPI), that maps equity ownership to corporate control, explicitly taking into account the possibility that centralized (proxy) voting behavior among dispersed shareholders converts fragmented ownership into corporate control. The NPI also takes into account the sequence of consolidated controls that may be channeled through an ownership network. We define a shareholder *i*’s NPI with respect to company *j* as the (*ex ante*) probability that *i* forms a coalition among other shareholders in *j* (either by consolidation or transitivity) which collects enough voting rights attached to their “combined” ownership ${\bar{x}}_{ij}$ to meet *j*’s quota *q*_*j*_. Formally, the NPI of the *ij* relationship is given as

$$\begin{array}{c} p_{ij}=\sum \mathrm{Pr}({\bar{y}}_{ij}=1|{\left\{{\bar{y}}_{ik}\right\}}_{i,k})\mathrm{Pr}\left({\left\{{\bar{y}}_{ik}\right\}}_{i,k}\right), \end{array}$$ (1)

where ${\left\{{\bar{y}}_{ik}\right\}}_{i,k}$ denotes the possible control linkages (both direct and indirect) from all the *i*s to all the *k*s upstream of *j* in the ownership network.

The NPI in the direct ownership network of Fig 1 is trivially obtained, as it amounts to the probability that *i* obtains direct, full control over *j*, or Pr(*y*_*ij*_ = 1). As we discussed above, none of the shareholders can alone prevail in *A*’s decision-making but each has equal chance of doing so. Thus, the NPI for each is given by *p*_*BA*_ = *p*_*CA*_ = *p*_*DA*_ = 1/3.

Eq (1) suggests that whether *i* gains control of *j* in the indirect ownership network depends on the structure of corporate control that *i* establishes upstream in the network. In Fig 2, if *C* obtains control of *D* (which occurs with probability 1/2) and consolidates the attached voting rights, it would fully control *A*; if *C* fails to control *D* with the complementary probability, then it could prevail in *A* with probability 1/3. Thus, *C*’s *network power* with respect to *A* is given by $p_{CA}=\frac{1}{2}\times 1+\frac{1}{2}\times \frac{1}{3}=\frac{2}{3}$ by consolidation. Once this coalition is realized, *C*’s NPI over *F* is given by $p_{CF}=\frac{1}{2}\times 1\times 1+\frac{1}{2}\times \frac{1}{3}\times 1=\frac{2}{3}$ by transitivity. If instead *E* controls *D* (again with probability 1/2), its individual NPI over *A* and *F* would be given by $p_{EA}=\frac{1}{2}\times \frac{1}{3}=\frac{1}{6}$ and $p_{EF}=\frac{1}{2}\times \frac{1}{3}\times 1=\frac{1}{6}$, respectively.

We also define *C*’s aggregated influence over all the companies at the global level. We write *i*’s *aggregate* NPI over all the *j*s in the entire network as *p*_*i*_ = ∑_*j*_ *p*_*ij*_. Letting *p*_*C*_ denote *C*’s aggregate NPI, we have $pC=pCC+pCD+pCA+pCF=1+\frac{1}{2}+\frac{2}{3}+\frac{2}{3}=\frac{17}{6}$ in Fig 2. Similarly, *E*’s *aggregate* NPI is given by $pE=pEE+pED+pEA+pEF=1+\frac{1}{2}+\frac{1}{6}+\frac{1}{6}=\frac{11}{6}$.

We note several qualifications about the NPI. First, the NPI quantifies shareholders’ *potential* to control corporate decision-making and it does not necessarily imply that they will actually exercise their power in practice. NPI does not assume that shareholders are rational decision-makers nor that they have complete information on the structure of network. NPI simply describes the power for shareholders to control corporate decision-making endowed by the structure of an ownership network. Second, the Shapley-Shubik power index is a special case of the *individual* NPI when it is applied to networks consisting only of direct ownership such as the one in Fig 1. This suggests that NPI can be considered as an extension of the Shapley-Shubik power index adapted for a complex corporate ownership structures that are often characterized by a myriad of cross-holdings, pyramids, and intermediate shareholders. In what follows, we refer to the power index for shareholder *i* in the context of direct ownership of *j* as the *Direct Power Index* (DPI), denoted by $p_{ij}^{D}$. Third, as a related issue, there is a concern that the Shapley-Shubik power index may not be adequate if there is a cycle in shareholding [4, 15]. We will discuss in the next section how our algorithm addresses this last concern.

## Numerical calculation method: Label propagation

Identifying corporate control in accordance with our model of the NPI is challenging. The structure of control over corporation *j* is conditional on the structure of control over intermediaries *k* that are upstream to *j* in an ownership network. The calculation of the NPI for each shareholder *i* over any corporations *j*, therefore, must take into account every possible path of control linkages through which *i*’s power of influence might extend to *j*. Furthermore, computing the exact value of NPI is difficult because cross-shareholdings cause cyclic ownership networks and because we assume the structure of control is realized probabilistically. We, therefore, propose a simulation-based approximation algorithm to calculate NPI, called *label propagation*.

The core algorithm, shown in Algorithm 1, consists of the following steps. For each company *j*, one of its shareholders *i* is randomly drawn and its voting rights are summed up. Continuing to add an additional shareholder *i* until the sum of their voting rights in a group reaches *j*’s quota, or $\sum {\bar{x}}_{ij}>q_{j}$, we record the last one that is added to the coalition as the “pivot” in *j*’s decision-making. The pivot controls *j*’s shares and associated voting rights (or ${\bar{y}}_{ij}=1$). After determining all of the pivots for every company *j* in the first step, the label for *j* (denoted by $L_{j}^{1}$) is replaced with *i*’s label (so that $L_{j}^{1}=i$). From the second step onward, the voting rights possessed by the shareholders under the unique pivots are summed up.

**Algorithm 1** Label propagation for NPI

**Input:** *N*, *x*_*ij*_, *q*_*j*_, *T*

**Output:** ${\hat{p}}_{ij}$

$L_{i}^{0}\leftarrow i$

**for** *t* = 1 to *T* **do**

  ${\bar{x}}_{ij}^{t}\leftarrow \sum_{k:L_{k}^{t-1}=i}x_{kj}$

 **for** *j* in *N* **do**

  *x* ← 0

  **while** *x* ≤ *q*_*j*_ **do**

   Randomly sample *i* from *N* without replacement.

    $x\leftarrow x+{\bar{x}}_{ij}$

  **end while**

  $L_{j}^{t}\leftarrow i$

 **end for**

**end for**

${\hat{p}}_{ij}\leftarrow \sum_{t=1}^{T}I\left\{L_{j}^{t}=i\right\}/T$

These steps are then repeated *T* times, and in each *t*th iteration every *j*’s label $L_{j}^{t}$ is updated, where *t* ∈ {0, …, *T*}. For *t* ≥ 2, if the label for any intermediate shareholders *k* is updated to *i* (or $L_{k}^{t}=i$), then their respective shares *x*_*kj*_ are consolidated and recorded as ${\bar{x}}_{ij}$ before returning to the first step.

After *T* iterations, we obtain the empirical distribution of $L_{j}^{t}$ which gives the frequency that company *j* is under *i*’s control. Let $I(L_{j}^{t}=i)$ denote a function that takes the value of one if $L_{j}^{t}=i$ and zero otherwise. Then, we approximate *i*’s *individual* NPI with respect to *j* such that:

$$\begin{array}{c} {\hat{p}}_{ij}\equiv \sum_{t=1}^{T}\frac{I(L_{j}^{t}=i)}{T}\approx p_{ij}, \end{array}$$ (2)

where ${\hat{p}}_{ij}$ denotes the approximate value of shareholder *i*’s NPI over company *j*. The approximated individual NPIs in our example of Fig 2 with *T* = 10, 000 iterations are ${\hat{p}}_{CD}=0.4936\approx \frac{1}{2}$, ${\hat{p}}_{ED}=0.5064\approx \frac{1}{2}$, ${\hat{p}}_{BA}=0.1662\approx \frac{1}{6}$, ${\hat{p}}_{CA}=0.6683\approx \frac{2}{3}$, ${\hat{p}}_{EA}=0.1655\approx \frac{1}{6}$, ${\hat{p}}_{BF}=0.1662\approx \frac{1}{6}$, ${\hat{p}}_{CF}=0.6683\approx \frac{2}{3}$ and ${\hat{p}}_{EF}=0.1655\approx \frac{1}{6}$, all of which are consistent with the theoretical values given in the previous section.

To ensure the reliability of our algorithm, we address three potential concerns. First, since we analyze the global ownership links among a much greater number of shareholders in a more complex network than analyzed before [7, 14], it takes many iterations in our algorithm for the ultimate owners’ *labels* to completely propagate down to all the target companies through the control linkages. In the very small example of Fig 2, ultimate owner *E*’s *label* reaches target company *F* after at least three iterations. Applying our algorithm to a data set consisting of many millions of shareholders, a large number of iterations will be needed to identify all the ultimate owners and their NPIs. This may result in some of companies carrying the “incorrect” label in early iterations. To address this potential problem, our algorithm discards the “propagation” results from early iterations $\bar{t}⪡T$.

Second, a cyclic structure in ownership networks presents a pervasive concern in calculating the concentration of corporate control [3–7]. In particular, when a circular shareholding structure is present due to cross-shareholding practices in the data set, our algorithm could overestimate the NPI for shareholders who are dominant in many corporations belonging to the same, large corporate group. A common strategy to address the problem of cycles is to adopt either a breadth-first-search (BFS) or a depth-first-search (DFS). We use neither. Instead, to circumvent this potential problem, our algorithm breaks loops by restoring the original label $L_{j}^{0}$ for some companies *j* with an arbitrary small probability. To see how this is done more concretely, consider cyclic ownership among some shareholders, where our algorithm above could erroneously identify a single shareholder or a group of shareholders as the ultimate owner(s) controlling all the shareholders involved and all of their downstream subsidiaries. This error would overestimate NPI for the estimate ultimate owner(s). We therefore avoid convergence on such a local solution by randomly initializing the label (i.e., the ultimate owner) for these shareholders.

Finally, calculation errors are inevitable in numerical approximation approaches. While there is no way of evaluating the accuracy of our calculation results with any objective criteria since the true values of the NPI are unknown, our algorithm yields fairly consistent approximated values with the theoretical predictions in our example above. Elsewhere, we have evaluated our algorithm’s calculation results for *T* < 20, 000 against the benchmark result with *T* = 20, 000 to find that the estimated NPIs converge to the benchmark values as the number of iterations increases [29].

## The power structure in the global ownership network

To analyze the structure of corporate control in the global ownership network, we use data from the Orbis database by *Bureau van Dijk* [30]. Our data set contains information on the ownership holdings in 49 million companies worldwide by 69 million shareholders (including individuals, corporations and governments alike) in 2016. For each of these shareholders, the Orbis database provides a unique identification number. Exploiting the unique IDs, we identify individual shareholders with multiple, separate investment institutions and treat them as the single entity.

The ownership network that we analyze here is not limited to publicly listed companies and transnational corporations or to specific regions; we also include in our analysis individuals and governments that own equity shares of corporations in the global economy regardless of their registered locations. Moreover, our analysis does not exclude any entity based on the size of ownership share in a target company, although previous studies often apply various share thresholds, including *x* ≥ 0.03 [23], *x* ≥ 0.05 [9, 18], and *x* ≥ 0.1 [7, 11]. Yet, our model and algorithm are intended to describe how a shareholder may obtain corporate control by consolidating the fragmented voting rights associated with dispersed ownership. Hence, in order to take into account the increasingly prevalent investment strategies such as index funds and ETFs, we follow a more recent practice [10, 14, 21] and include in our analysis any *x* > 0 recorded in the Orbis database. The resulting global ownership network for our analysis consists of over 65 million nodes, of which about 37 million are ultimate owners, and over 90 million ownership linkages.

Applying our algorithm to this data set, we calculate the *individual* NPI for each shareholder *i* with respect to a specific target company *j*, as well as the *aggregated* NPI for each investor. The *aggregate* NPI measures the overall power to control corporations in the global ownership network. Our estimated values of shareholder *i*’s aggregated NPI can usefully be interpreted as the expected number of companies under direct or indirect control of *i* in the entire global ownership network. In addition, we use target company *j*’s revenue *v*_*j*_ to calculate the weighted NPI for the aggregated level, denoted by *vp*_*i*_ = ∑_*j*_ *v*_*j*_ *p*_*ij*_. In the rest of this section, we characterize the structure of corporate control using *aggregate* NPIs, as compared to other measures.

Since our goal is to measure the distribution of power among the shareholders to fully control managerial decision-making of target companies in the global ownership network, we select 50% of voting-rights as the cutoff to identify corporate control. This selection is consistent with the previous studies that intend to describe formal, full control [14, 20].

### Comparing the NPI to the DPI

Table 1 lists the shareholders in the top twenty of the DPI values weighted by their operating revenue (in U.S. billion dollars) and aggregated at the global level, $vp_{i}^{D}$. This value can be interpreted as the expected value of total operating revenues that a shareholder *i* may be able to directly control in the global economy. For comparison, the table also includes their weighted NPI that is also aggregated at the global level. Table 2 lists the top-20 shareholders in terms of their (weighted, aggregated) NPI values. Comparing these two tables elucidates the different landscapes of corporate control as seen with direct ownership alone versus indirect, networked ownership.

**Table 1:** Shareholders with Top-20 DPI in 2016.

	Rank (DPI)	Rank (NPI)	DPI^(*2)^	NPI^(*3)^
			$v{\hat{p}}_{i}^{D}$	$v{\hat{p}}_{i}$
BlackRock Inc	1	31340	1479.35	0.26
Vanguard Group Inc	2	360167	1228.08	0.01
SASAC ^(*1)^	3	-	809.30	0
Fujitsu Ltd.	4	-	728.61	0
State Street Corp.	5	3297262	715.24	0.00
Royal Dutch Shell PLC	6	-	590.24	0
Capital Group Co Inc	7	3	564.92	2432.02
China National Petroleum Corporation	8	-	520.22	0
China Petrochemical Co., Ltd.	9	-	513.46	0
Fidelity Management and Research LLC	10	4262527	504.78	0.00
Bidvest Group Ltd	11	-	487.79	0
Walton Family	12	14	482.13	828.96
Government of Norway	13	2	453.13	2617.73
Exxon Mobil Corp	14	30	443.66	485.35
Government of Saudi Arabia	15	28	387.30	535.48
JPMorgan Chase & Co	16	1989990	371.15	0.00
China Petroleum & Chemical Corp	17	-	368.86	0
ENGIE	18	35	361.14	419.77
Toyota Motor Corporation	19	-	304.49	0
Allianz SE	20	23	298.29	584.49

^(*1)^ State-owned Assets Supervision and Administration Commission of the State Council.

^(*2)^ DPI: Aggregate DPI weighted by operating revenue in U.S. billion dollars.

^(*3)^ NPI: Aggregate NPI weighted by operating revenue in U.S. billion dollars.

**Table 2:** Shareholders with Top-20 NPI in 2016.

	Rank (NPI)	Rank (DPI)	NPI^(*3)^	DPI^(*2)^
			$v{\hat{p}}_{i}$	$v{\hat{p}}_{i}^{D}$
Government of China^(*4)^	1	272	7392.64	57.14
Government of Norway	2	13	2617.73	453.13
Capital Group Co. Inc.	3	7	2432.02	564.92
Wellington Management Group LLP	4	33	2050.74	240.93
Government of South Africa	5	627	1787.87	29.68
Vanguard Index Funds	6	432469	1479.67	0.01
Sun Life Financial Inc.	7	50	1166.81	199.41
Government of the Russian Federation	8	84	1049.08	132.73
Johnson Family	9	10717	1043.85	1.41
Sumitomo Mitsui Trust Holdings, Inc	10	96	989.42	124.91
Mitsubishi UFJ Financial Group Inc	11	88	960.78	129.56
HSBC Custody Nominees (Australia) Ltd	12	89	951.09	128.81
Vanguard Bond Index Funds	13	666167	856.39	0.01
Walton Family	14	12	828.96	482.13
Familien Porsche/Piech	15	2049428	714.38	0.00
Dimensional Fund Advisors LP	16	26	686.24	268.43
Government of France	17	40	643.60	228.21
Government of South Korea	18	62	640.95	169.42
Government of Singapore	19	498	640.49	35.02
TIAA Board of Overseers	20	598	637.93	30.00

^(*2)^ DPI: Aggregate DPI weighted by operating revenue in U.S. billion dollars.

^(*3)^ NPI: Aggregate NPI weighted by operating revenue in U.S. billion dollars.

^(*4)^ Unlike [14], our calculation does not include Hong Kong in the People’s Republic of China.

We make two observations about Table 1. First, the majority of the shareholders with top-20 DPI consists either of energy companies and financial institutions. Six of top-20 DPI shareholders are fossil-oil companies (Royal Dutch Shell, Exxon Mobil, ENGIE, and three large Chinese energy corporations), and six other highest DPI holders are financial institutions. In particular, BlackRock, Vanguard, and State Street—the Big Three asset managers—are among the top five shareholders in terms of their capacity to directly control corporate decision-making.

It is interesting to note that our calculation result for the top DPI in 2016 is similar to previous work that emphasizes financial institutions as control-holders. Vitali et al. [7] report that the financial companies holding the top 3 total network control in 2007 are Fidelity Management & Research, Capital Group, and BlackRock [7], while Brancaccioa et al. [9] find that Vanguard Group, BlackRock, and Fidelity are among the top 3 control-holders in 2016 [9]. Our analysis of the DPI also finds all of these institutions in the top-20 list (Table 1). Moreover, our power index (DPI) is consistent with the evidence reported by Fichtner et al. regarding these asset managers collectively constituting the largest shareholder in at least 40% of all U.S. listed companies [8]. The consistency of the results with the recent studies gives face validity to our power index.

The second observation about Table 1 is that the most top-20 DPI shareholders have either a very small or no NPI value. Eight of them do not have their own NPI since these shareholders are owned by other shareholders upstream in the investment flow. SASAC, for example, is owned and controlled by the Government of China (PRC) that turns out to be most influential measured in terms of the NPI as shown in Table 2. Similarly, many major financial institutions have minuscule NPI values because they are in theory subject to control by their shareholders upstream. For instance, JPMorgan Chase & Co is owned by 116 shareholders, where top-three (direct, not indirect) owners are BlackRock Inc with 6.28% share, Vanguard Group Inc with 5.28%, and State Street Corp with 4.17%. However, these blockholders of JPMorgan Chase have relatively weaker networked power of corporate control. BlackRock Inc., the highest DPI-holder, is ranked the 31,340th in terms of the NPI, as its ownership in turn is held by blockholders: three largest shareholders are PNC Bancorp Inc with 21.12% ownership, Norges Bank with 7.21%, and Government Pension Fund of Norway with 5.76%. Similarly, Vanguard Group Inc.’s NPI value is also small because Vanguard Index Funds and Vanguard Bond Index Funds respectively hold its 25% and 10% ownership, both of which are among the top-20 NPI holders in Table 2.

These results regarding the gap between DPI and the NPI values have three important implications. First, some ultimate owners can obtain the power of corporate control much larger than the size of their direct equity ownership, by extending their influence through the network and sometimes absorbing the power of corporate control held by their intermediaries. The second implication has to do with the interpretation of the NPI. Namely, the NPI evaluates a shareholder’s *potential* power structurally induced by their relative positions in the ownership network. Our model of NPI remains agnostic as to whether those ultimate owners actually exercise their power to control companies. The NPI alone does not tell us whether the Government of China takes advantage of its indirect control over China National Petroleum Corporation or whether Norges Bank and the Norwegian Government Pension Fund work together to influence BlackRock Inc. Nor does it tell us whether intermediate, downstream shareholders (such as Vanguard Group Inc) are free to exert their own influence independently of (or on behalf of) their upstream owners (such as Vanguard Index Funds).

The last but not least important implication is that financial institutions may not be able to control the world economy as much as previously suggested in terms of who has the final say on managerial control in the global ownership network. What emerges as more powerful control-holders than financial institutions are state governments. Indeed, seven of the top-20 shareholders in terms of *network power* are sovereign governments (Table 2). In particular, the government of the People’s Republic of China has an exceptionally high NPI values, nearly three times greater than that of Norway.

The governments tend to have a large gap between the indirect and direct control. This suggests that we may underestimate sovereign governments’ capacity to control corporate decision-making around the world if we rely solely on the disclosed balance sheet. Unless we take into account how shareholders can channel their influence through the network, the governments’ capacities to control the global economy remain rather invisible. Although it is beyond the scope of this article to explore the reason why this is happening, our analysis indicates that some sovereign governments possess a large but hidden capacity to control corporate activities through the global network.

### Comparing the NPI to *network control*

Vitali et al.’s *network control* quantifies the value of full control a shareholder *i* obtains in a sequence of direct controls from *i* to *k* and from *k* to *j*. The difference between our concept of NPI and *network control* manifests in two aspects: (1) the consolidation of corporate control and (2) corporate control by intermediaries.

**Fig 3:**
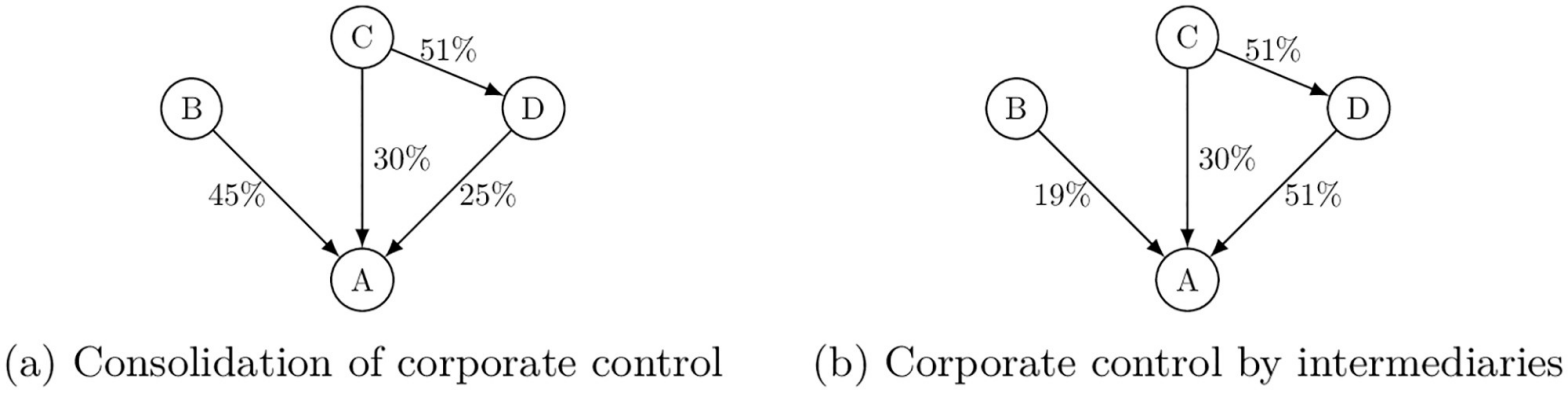
Comparison between NPI and *network control*.

As for the first difference, the NPI describes how a shareholder may consolidate ownership shares to obtain the power of corporate control, while a shareholder does not consolidate corporate control under the concept of *network control* unless the shareholder directly controls a target company whose voting rights the shareholder could otherwise consolidate. To see this more concretely, suppose a simple ownership network in Panel (a) of Fig 3. Since shareholder *C* has a simple majority of voting rights in *D*, its *individual* NPI with respect to *D* is *p*_*CD*_ = 1. Then, consolidating *D*’s 25% ownership shares in *A* and its own 30% shares, *C* obtains full control in *A* so that *p*_*CA*_ = 1 as shown in the matrix of individual NPIs in Eq (3). Ultimate owners, *B* and *C*, also control themselves: *p*_*BB*_ = 1 and *p*_*CC*_ = 1.

$$\begin{array}{c} p=(\begin{array}{cccc} 0 & 0 & 0 & 0\\ 0 & 1 & 0 & 0\\ 1 & 0 & 1 & 1\\ 0 & 0 & 0 & 0 \end{array}) \end{array}$$ (3)

With the concept of *network control*, on the other hand, *C*’s full control of *D* does not allow *C* to consolidate *D*’s 25% ownership in *A* and its own 30% shares in *A* to obtain full control in *A* even though *C* would otherwise control a majority of voting rights in *A* (i.e., ${\bar{x}}_{CA}=x_{CA}+x_{DA}=0.55$). Among three models Vitali et al. [7] use to map ownership shares to corporate control, we focus on their “threshold model,” where a shareholder *i* obtains full control in *j* if *x*_*ij*_ > *q*_*j*_ = 0.5. Then, this example gives the *network control* matrix as shown in Eq (4), where $c_{CD}^{\text{net}}=1$ but $c_{CA}^{\text{net}}=0$. The only way for *C* to seize corporate control in *A* would be to establish the sequence of control by transitivity. This would be possible if *D* had a majority of shares in *A* (i.e., *x*_*DA*_ ≥ *q*_*A*_) as shown in Panel (b) of Fig 3.

$$\begin{array}{c} c^{\text{net}}=(\begin{array}{cccc} 0 & 0 & 0 & 0\\ 0 & 0 & 0 & 0\\ 0 & 0 & 0 & 1\\ 0 & 0 & 0 & 0 \end{array}) \end{array}$$ (4)

This last point leads to another difference between two concepts. While the NPI is intended to characterize the ultimate owners’ power of corporate control, the concept of *network control* allows intermediaries to have some degree of control. Consider the example in Panel (b) of Fig 3. Shareholder *D* is an intermediary in *C*’s control chain with respect to *A*. Shareholder *C* is an ultimate owner who has the power to directly control *D* and to indirectly control *A* through its control in *D*, so that the NPI matrix shown in (5) remains the same as in Eq (3). On the other hand, this example changes the *network control* matrix as shown in Eq (6). Since with *network control* intermediary *D* is allowed to have its own control independently of its upstream shareholder, *D*’s simple majority in *A* gives $c_{DA}^{\text{net}}=1$, in addition to its upstream shareholder *C*’s control in *A* that is channeled through intermediary *D* so that $c_{CA}^{\text{net}}=1$. In this sense, *network control* double-counts corporate control over *A*’s decision-making among the shareholders, so that $\sum_{j}c_{ij}^{\text{net}}>1$ for *j* = *A* as shown in Eq 6. This leads to overestimation of the power of corporate control in the presence of cross-sharing.

$$\begin{array}{c} p=(\begin{array}{cccc} 0 & 0 & 0 & 0\\ 0 & 1 & 0 & 0\\ 1 & 0 & 1 & 1\\ 0 & 0 & 0 & 0 \end{array}) \end{array}$$ (5)

$$\begin{array}{c} c^{\text{net}}=(\begin{array}{cccc} 0 & 0 & 0 & 0\\ 0 & 0 & 0 & 0\\ 1 & 0 & 0 & 1\\ 1 & 0 & 0 & 0 \end{array}) \end{array}$$ (6)

Now that we have clarified the theoretical difference between the NPI and *network control*, we turn to empirical comparisons. We calculate the *network control* value for the ultimate owners weighted by their respective operating revenues using the “threshold model.” Table 3 lists the top 20 ultimate owners in terms of *network control* along with the NPI if applicable.

**Table 3:** Ultimate owners with Top-20 “Network Control” in 2016.

	Rank (*c*^net^)	Rank (NPI)	*c*^net^^(*4)^	NPI^(*3)^
				$v{\hat{p}}_{i}$
Government of China	1	1	6199.53	7392.64
Royal Dutch Shell PLC	2	-	1239.85	0
Government of the Russian Federation	3	8	948.50	1049.08
Walton Family	4	14	836.02	828.96
Fujitsu Ltd	5	-	779.61	0
BP PLC	6	-	688.28	0
Familien Porsche/Piech	7	15	641.37	714.38
Total S.A.	8	-	530.07	0
Bidvest Group Ltd	9	-	527.14	0
Vitol Holding II SA	10	29	510.86	504.90
Wesfarmers Ltd	11	-	493.92	0
Exxon Mobil Corp	12	-	489.54	485.35
Toyota Motor Corp	13	-	466.99	0
Government of Saudi Arabia	14	28	457.41	535.48
Glencore PLC	15	-	425.36	0
Daimler AG	16	-	420.13	0
Government of France	17	17	419.14	643.6
Berkshire Hathaway Inc	18	751045	414.28	0
E.ON SE	19	34	412.78	423.02
ENGIE	20	35	400.19	419.77

^(*3)^ NPI: Aggregate NPI weighted by operating revenue in U.S. billion dollars.

^(*4)^ *c*^net^: Aggregate Network Control weighted by operating revenue in U.S. billion dollars.

The most significant difference with top-20 NPI shareholders (Table 2) is that financial institutions are not listed. While JP Morgan is ranked 25th (thus not shown in the table), the Big Three asset managers—BlackRock, Vanguard Group Inc. and State Street Corp.—are ranked 882nd, 96,635th and 485th respectively. As noted above, these large financial institutions only have small or no NPI values because they are intermediaries owned by other ultimate owners. On the other hand, one would hypothesize that these financial institutions have large *network control* values because this concept, unlike the NPI, allows intermediaries like these financial institutions to hold their own control. Yet, the financial institutions turn out to have relatively smaller *network control* values (Table 3). We interpret that these results illustrate how *network control* underestimates the potential influence derived from fragmented voting rights attached to dispersed ownership.

Instead, according to this table, *network control* picks up the influence of three other types of shareholders. First, governments with state-owned corporations, especially China, Russia and Saudi Arabia, are evaluated as having high *network control* values. This result may reflect the fact that authoritarian governments often fully control the management of state-owned corporations that typically are utility companies and banks with large revenues [10]. A similar observation can be made for the Walton Family that owns and controls Walmart, the world’s largest company by revenue.

Second, six among the top-20 shareholders with high values of *network control* are energy & oil companies, of which four—Royal Dutch Shell, British Petroleum (BP), France’s Total, Exxon Mobil—are among the six Maritime Oil Majors. Third, four manufacturing companies—Fujitsu, Porsche, Toyota Motors, and Daimler—are also among the top 20, reflecting the fact that each owns many companies on its supply chain as subsidiaries. These oil and manufacturing companies, however, do not have NPI values because they are owned by other companies.

### Comparing the NPI to NSR

Recall that NSR, which is similar to the concept of “integrated ownership” presented by Brioschi et al. [12], shows the the sum of equity capitals of firms a shareholder owns directly and indirectly in the ownership network. On the other hand, NPI respresents the potential for corporate control. The difference between the capacity to control corporate decision-making and the ownership of liquidity stakes evidently manifests in the comparison between NPI and NSR. In particular, the value of NPI in excess of the fair share suggested by NSR can be interpreted as the degree to which control of capital goes beyond the limits of the ownership of equity stakes, resulting in the concentration of voting power even under the one-share, one-vote rule.

**Fig 4:**
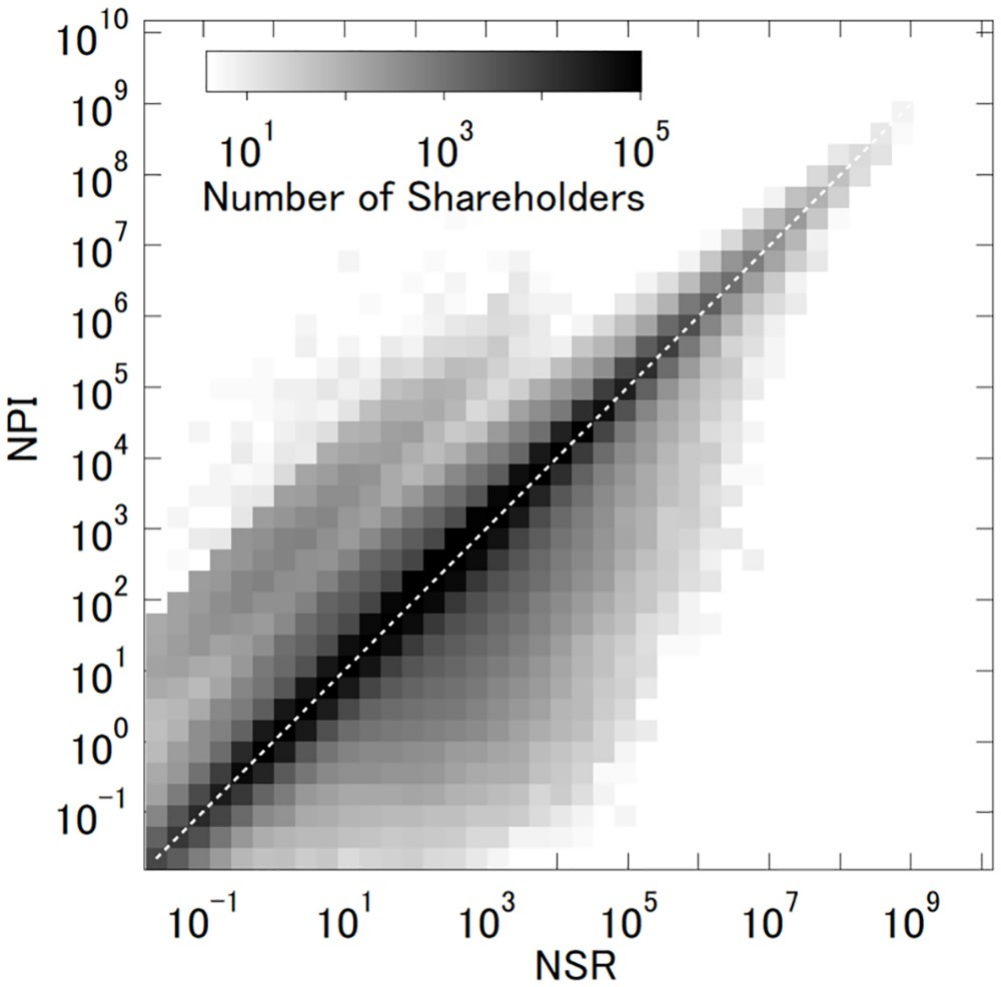
Comparison of aggregate NPIs with NSRs.

The heatmap in Fig 4 shows the density of shareholders, with the corresponding NSR and NPI values on the horizontal and vertical axes respectively. The shareholders with the value of 0.01 or less are not shown for either NSR or NPI.

We make two observations in this comparison. First, while the 45-degree line represents the cases where the equity ownership linearly translates into control in the target company, more shareholders are located below this line. This implies that more shareholders appear to have greater influence in terms of NSR than they do in terms of NPI. This result is consistent with our intuition because while NSR linearly evaluates the share of equity stakes regardless of the size of shareholding, NPI has a nonlinear tipping-point effect. That is, NPI discards a shareholder’s shares if its voting rights fail to (collectively) form a winning coalition in the target company *j*, resulting in a nonlinear gap between the size of shareholding and the size of network power. In other words, since NPI measures the shareholders’ power to control corporate decisions, the divergence of NSR from NPI corresponds to the equity stakes that are not converted into corporate control. Relatively high NSR values that fail to generate equally high NPI values are concentrated in the range between 10^1^ and 10^5^.

Second, the shareholders with sufficiently large values of both NPI and NSR (e.g., >10^7^) in the northeast corner tend to be distributed symmetrically around the 45-degree line, suggesting that the shareholders with exceptionally high values in NSR successfully obtain a high capacity to control corporate governance through the network, as reflected in NPI values. This is evident in the list of top-20 shareholders as measured by NSR in Table 4. Except for one individual (i.e., Mr. David Booth) and two firms, these top-20 NSR shareholders also have high NPI values, efficiently converting their equity shares to the network power of corporate control.

**Table 4:** Shareholders with Top-20 NSR in 2016.

	Rank (NSR)	Rank (NPI)	NSR^(*5)^	NPI^(*3)^
				$v{\hat{p}}_{i}^{N}$
Government of China	1	1	6264.22	7392.64
Government of Norway	2	2	4141.90	2617.73
Johnson Family	3	9	2606.42	1043.85
Wellington Management Group LLP	4	4	2586.33	2050.74
Vanguard Index Funds	5	6	2344.99	1479.67
Everwin Company Limited	6	6319	1487.21	1.76
Japan Trustee Services Bank Ltd	7	22	1184.96	586.05
Mr. David Booth	8	11832429	1154.71	0.00
Government of South Africa	9	5	1144.62	1787.87
Master Trust Bank of Japan Ltd	10	25	1118.21	576.91
Vanguard Bond Index Funds	11	13	1067.34	856.39
Government of South Korea	12	18	986.37	640.95
Government of the Russian Federation	13	8	901.32	1049.08
TIAA Board of Overseers	14	20	841.38	637.93
Government of Singapore	15	19	756.14	640.49
Government of France	16	17	709.88	643.60
Government of Qatar	17	37	688.60	388.52
BNP Paribas	18	33	647.74	460.62
Capital World Investors	19	31	635.07	481.42
Geode Holdings Trust	20	11832460	613.55	0.00

^(*3)^ NPI: Aggregate NPI weighted by operating revenue in U.S. billion dollars.

^(*5)^ NSR: Aggregate NSR weighted by operating revenue in U.S. billion dollars.

The comparison of NPI to NSR also has an implication for the agency problem in the corporate finance literature. La Porta et al.’s [11] seminal work on corporate control asks “whether the cash flow ownership rights of the controlling shareholders are substantially different from their voting rights,” and they finds that controlling shareholders in large companies have power significantly in excess of their cash flow rights [11]. Our result, however, indicates that that is not the case at least for *very* large corporations as shown in Table 4.

This, nonetheless, does not suggest that recent corporate governance is free from the principal-agent problem. In fact, the conflict between controlling shareholders and minority shareholders may be more pervasive than we have assumed. On the one hand, as Fig 4 indicates, more shareholders have less controlling rights than their equity shares. On the other hand, Tables 2 and 4 show that state governments, along with financial institutions, are well represented among the ultimate owners with controlling rights in excess of their equity rights. This implies that the agency problem exists not only in the traditional context of corporate governance, but also in a more political context. If a corporate in a key industry happens to be controlled by a foreign government with geo-strategic rivalry, the agency problem implies a significant political risk not only for corporation shareholders but also for the wider stakeholders [14] as we witness in the concerns expressed by the European Commission related to foreign takeover [31].

## Conclusion

This article makes three contributions. First, we propose the *network power index* (NPI) to measure the influence that each investor *i* has on any specific company *j* in a networked society. The NPI allows us to capture the indirect power that *i* has over *j* by controlling the influence that a third-party entity *k* has on *j*. This *i* → *k* → *j* indirect influence has been overlooked in the past applications of the Shapley-Shubik power index because they only capture direct influence. Second, because calculating the NPI values entails indeterminacy in the presence of cyclic ownership structure, we propose an algorithm to numerically calculate NPI values. Third, applying our method and algorithm to data on global shareholding networks, we show that the gap between NPI and other measures of corporate control are substantial. This suggests that NPI allows us to identify “hidden influencers’’—including sovereign governments—in our globalized *political* economy.

Since the objectives of this article have been to propose a theoretical model and algorithm to measure the power of corporate control in ownership networks, refinements of empirical analysis are needed in several areas to better describe the actual corporate control around the world. First, we used the simple majority rule as the quota in the Shapley-Shubik-esque weighted voting game framework, while the empirical literature has adopted various other levels ranging from 5% to 25% in a simple cutoff model. In reality, there are abundance of instances where a shareholder with consolidated voting rights of less than 50% exercising effective control. Relatedly, there are multiple classes of shares including non-voting shares and “golden shares” alike. Our analysis strictly adopts the one-share one-vote rule. Future research should relaxed these assumptions, taking into account complex practices around the world.

Second, the corporate finance literature emphasizes the importance of legal institutions as the determinants both of the structure of corporate control, such as investor protection and product and labor regulations. For example, La Porta et al. [11] suggest that countries with poor shareholder protection tend to have more large firms controlled by the state government. To accurately describe the corporate ownership, therefore, future empirical analysis would need account for country variations in the investment environment. Lastly, the study on algorithms in identifying control in the ownership network is also needed. As the sample size on ownership available for analysis is rapidly increasing, a computational challenge has forced the scholars to restrict their analysis to a subset of the sample. Limiting the analysis to a subset of global ownership network not only makes it difficult to compare findings across various studies, but also increases the risk of introducing biases in causal inference. To address this potential threat to scientific inquiry into corporate control and its implications for international political economy, it is important to devise more efficient algorithms in this area.
